# Supplementary material for: The association between adolescents’ independent food purchasing and dietary quality differs by socioeconomic status: Findings from a pilot study
Source: PLoS One. 2025 Sep 4;20(9):e0312903. doi: 10.1371/journal.pone.0312903 (PMC12410734; doi:10.1371/journal.pone.0312903)
Supplement: S1 Table — (DOCX) [file pone.0312903.s001.docx]

**S1 Table. Coding of food purchases against the UK Eatwell Guide**

| **Coding food purchases against Eatwell Guide** | | |
| --- | --- | --- |
| **Foods/drinks categorised as adhering to the Eatwell Guide** | **Foods/drinks categorised as not adhering to the Eatwell Guide** | **Combination/uncategorised foods/drinks** |
| - Bread - Breakfast cereal (No added sugar) - Fermented milk drink - Fruit - Fruit Juice - Fruit Smoothie - Non-sugar sweetened carbonated drink - Milk - Nuts - Pasta/rice - Sushi - Tea/Coffee - Steak - Roasted chicken - Vegetables - Water | - Battered Fish - Biscuits - Burgers/kebabs - Cakes - Chicken nuggets/battered chicken - Chips/Fries - Chocolate confectionary - Crisps - Energy drinks - Ice-cream - Iced Tea/ Iced Coffee/ Bubble Tea - Milkshakes - Non-carbonated sugar sweetened drinks - Popcorn - Pizza - Processed meat (chorizo) - Ready meals - Sauces and dips - Sugar confectionary - Sausage roll, pasty, pie - Sugar sweetened carbonated drinks - Sweet pastries | - Sandwich or wrap - Other hot drink - Dumplings |
