# Supplementary material for: The association between adolescents’ independent food purchasing and dietary quality differs by socioeconomic status: Findings from a pilot study
Source: PLoS One. 2025 Sep 4;20(9):e0312903. doi: 10.1371/journal.pone.0312903 (PMC12410734; doi:10.1371/journal.pone.0312903)
Supplement: S2 Table — Linear regression results for association between food purchasing (exposure) and diet quality (SDs) (outcome). (DOCX) [file pone.0312903.s002.docx]

**S2 Table.** **Sensitivity analysis when purchases coded as ‘0’ are treated as missing.** Linear regression results for association between food purchasing (exposure) and diet quality (SDs) (outcome)

| **Exposure variable** | **Unadjusted β  (95% CI)** | **SE** | **p-value** | **Adjusted β  (95% CI)^*^** | **SE** | **p-value** |
| --- | --- | --- | --- | --- | --- | --- |
| Number of weekly purchases | -0.00  (-0.04, 0.04) | 0.02 | 0.94 | 0.00  (-0.04, 0.05) | 0.02 | 0.85 |
| Weekly Purchasing Healthfulness Score (healthfulness units) | 0.43  (-0.01, 0.87) | 0.22 | 0.06 | 0.34  (-0.13, 0.81) | 0.24 | 0.16 |
| ^*^Models adjusted for age, gender, ethnicity, and household SES.  Purchasing Healthfulness Scores closer to 1 indicate healthier purchasing  *In recognition that scoring food purchases categorised as ‘mixed’ as ‘0’ may not be capturing the missing information associated with these foods, a sensitivity analysis was conducted with these purchases treated as missing. This sensitivity analyses showed no impact on the overall study findings.* | | | | | | |
