# Supplementary material for: The association between adolescents’ independent food purchasing and dietary quality differs by socioeconomic status: Findings from a pilot study
Source: PLoS One. 2025 Sep 4;20(9):e0312903. doi: 10.1371/journal.pone.0312903 (PMC12410734; doi:10.1371/journal.pone.0312903)
Supplement: Attachment — Submitted filename: Shaw_PEER REVIEW response_May25_F2.docx [file pone.0312903.s003.docx]

Editor-in-chief

PLOS One

28^th^ May 2025

Dear Editor,

**RE: PONE-D-24-45297
What role do adolescents’ independent food purchasing choices play in their dietary quality?**

We thank the editorial team and reviewer for their helpful review of our manuscript and for providing us with valuable feedback. Below, we respond in detail to the points raised and have attached a revised version of our manuscript with changes highlighted in red font. As requested, a clean version with the amendments has also been attached.

Below, we have reproduced the reviewers’ comments in **bold** and added our responses in regular font indicating how we have altered the paper to address the comments. Excerpts from the manuscript are also provided in *italicised* text with corresponding page and line numbers for the tracked manuscript, and text changes highlighted in red font.

We look forward to hearing the outcome of the peer review process.

Yours sincerely,

The authors

**Journal Requirements:**

**When submitting your revision, we need you to address these additional requirements.**

1. **Please ensure that your manuscript meets PLOS ONE's style requirements, including those for file naming. The PLOS ONE style templates can be found at** [**https://journals.plos.org/plosone/s/file?id=ba62/PLOSOne_formatting_sample_title_authors_affiliations.pdf**](https://journals.plos.org/plosone/s/file?id=ba62/PLOSOne_formatting_sample_title_authors_affiliations.pdf)

The required edits have now been made to the manuscript to ensure it meets PLOS ONE’s style requirements.

**2. We note that the grant information you provided in the ‘Funding Information’ and ‘Financial Disclosure’ sections do not match. When you resubmit, please ensure that you provide the correct grant numbers for the awards you received for your study in the ‘Funding Information’ section.**

The funding information has been checked and corrected to address inconsistencies. Please see the revised wording below. Corresponding changes have been made in the online submission system under ‘Funding Information’ however, we are unable to edit the ‘Financial disclosure’ statement. We would appreciated if this could be done by the editorial team.

*This work was supported by the National Institute for Health Research Southampton Biomedical Research Centre (PhD studentship), the UK National Institute for Health Research Programme Grants for Applied Research (RP-PG-0216-20004) and the UK Medical Research Council (MC_UU_12011/4). The views expressed in this publication are those of the author(s) and not necessarily those of the National Health Service, the National Institute for Health Research and the UK Department of Health and Social Care. The funders had no role in study design, data collection and analysis, decision to publish, or preparation of the manuscript.*

**3. Thank you for stating the following financial disclosure: [This research and the authors of this paper are supported by the following funding sources: National Institute for Health Research Southampton Biomedical Research Centre; UK National Institute for Health Research Programme Grants for Applied Research (RP-PG-0216-20004); and UK Medical Research Council (MC_UU_12011/4). The views expressed in this publication are those of the author(s) and not necessarily those of the National Health Service, the National Institute for Health Research, and the UK Department of Health and Social Care.]. Please state what role the funders took in the study. If the funders had no role, please state: "The funders had no role in study design, data collection and analysis, decision to publish, or preparation of the manuscript." If this statement is not correct you must amend it as needed. Please include this amended Role of Funder statement in your cover letter; we will change the online submission form on your behalf.**

The funding disclosure has now been edited in describe the role of the funder. Please see excerpt below.

*This work was supported by the National Institute for Health Research Southampton Biomedical Research Centre (PhD studentship), the UK National Institute for Health Research Programme Grants for Applied Research (RP-PG-0216-20004) and the UK Medical Research Council (MC_UU_12011/4). The views expressed in this publication are those of the author(s) and not necessarily those of the National Health Service, the National Institute for Health Research and the UK Department of Health and Social Care. The funders had no role in study design, data collection and analysis, decision to publish, or preparation of the manuscript.*

**4. Thank you for stating the following in your Competing Interests section: [SS, SC, CC, DS, MB have no conflicts of interests to declare. CV has a non-financial research collaboration with a UK supermarket chain. The study described in this manuscript is not related to this relationship.]. Please complete your Competing Interests on the online submission form to state any Competing Interests. If you have no competing interests, please state "The authors have declared that no competing interests exist.", as detailed online in our guide for authors at http://journals.plos.org/plosone/s/submit-now This information should be included in your cover letter; we will change the online submission form on your behalf.**

We have now added our competing interests to the cover letter for this resubmitted manuscript. Thank you for entering these details in the online submission form on our behalf.

**5. In the online submission form, you indicated that [Due to ethical restrictions imposed in the interest of protecting participant confidentiality, the data underlying this study are available upon request. Researchers wishing to use the data can make apply to the research team by emailing mrcleu@mrc.soton.ac.uk. Subject to approval that the intended purpose is compatible with the study’s ethical approval and formal agreements regarding confidentiality and secure data storage being signed, the data would then be provided.].**

**All PLOS journals now require all data underlying the findings described in their manuscript to be freely available to other researchers, either 1. In a public repository, 2. Within the manuscript itself, or 3. Uploaded as supplementary information.**

**This policy applies to all data except where public deposition would breach compliance with the protocol approved by your research ethics board. If your data cannot be made publicly available for ethical or legal reasons (e.g., public availability would compromise patient privacy), please explain your reasons on resubmission and your exemption request will be escalated for approval.**

Ethical restrictions are placed on the sharing of this data to ensure anonymity of the study participants who are aged 18 years and under. During the consent process, participants aged 16 and over, or the parents of participants under 16, gave permission for their data to be used in ethically approved research only. In addition, they provided permission for their data to be shared exclusively with approved researchers. This consent process was approved by the University of Southampton Faculty of Medicine ethic committee (ERGO 57044, contact [rgoinfo@soton.ac.uk](mailto:rgoinfo@soton.ac.uk)).

The anonymised data used in this study are available to bone fide researchers on request from the University of Southampton. Researchers wanting to access these data should contact [**mrcleu@mrc.soton.ac.uk**](mailto:mrcleu@mrc.soton.ac.uk) and declare their suitability to the criteria for accessing confidential data. Please see the amended data availability statement below, this has been updated in the submissions form.

Data availability statement

*Data cannot be shared publicly because of consent and ethical restrictions imposed in the interest of protecting the* anonymity *of the study participants who are aged 18 years and under. Data are available from the University of Southampton (contact via* [***mrcleu@mrc.soton.ac.uk***](mailto:mrcleu@mrc.soton.ac.uk)*) for researchers who meet the criteria for access to confidential data.*

**6. Please amend your list of authors on the manuscript to ensure that each author is linked to an affiliation. Authors’ affiliations should reflect the institution where the work was done (if authors moved subsequently, you can also list the new affiliation stating “current affiliation:….” as necessary).**

The list of authors has now been amended and include all authors’ affiliations.

**7. We note that you have referenced (unpublished data) on page 9, which has currently not yet been accepted for publication. Please remove this from your References and amend this to state in the body of your manuscript: (ie “Bewick et al. [Unpublished]”) as detailed online in our guide for authors** <http://journals.plos.org/plosone/s/submission-guidelines#loc-reference-style>

A reference is now available for this work and has been included in the manuscript.

**Additional Editor Comments:**

**Methodology
1. The authors should indicate which of the variables were normally distributed and which ones were not. It will also be helpful in the variables section for the authors to indicate how the variables were categorized. For example, readers are lost as to how the diet quality finally used in the analysis.**

Details to describe the variable that is normally distributed, and those which were not, have now been added to the manuscript.

*Page 11, lines 230-232*

*The normally-distributed continuous variable (diet quality) was summarised using mean (SD). Non-normally distributed continuous variables (age, individual SES, household SES) were summarised using median (IQR).*

Details have also been added to the manuscript to describe how the diet quality score was used in the analysis.

*Pages 10-11, lines 220- 228*

*Using a 20-item FFQ, participants recorded how frequently they consumed each food group over the previous month. These dietary data were used to calculate a diet quality score for each participant following published methodology specifically developed to assess diet quality among UK adolescents (Shaw et al. 2021). This diet quality score has been validated against fourteen nutritional biomarkers, including serum folate, homocysteine, total carotenoids and vitamins B12, C and D (Shaw et al. 2021). Diet quality scores were used in analyses as a continuous variable with higher scores represent better quality diets consistent with the UK dietary guidelines.*

**2. In table 5, it will be good to include the standard errors because the confidence intervals are quite wide. With a sample of size of 80 and some missing participants, the authors should include a section about their regression diagnostics.**

We have considered this comment and decided not to include the standard errors in the results tables because these can be calculated from the confidence intervals already presented. For example, the upper bound of the confidence interval is β + (1.96 x SE). We have added a sentence in the methods section to describe how the standard errors can be calculated.

Page 12, lines 257-258

*Regression model results are presented with effect sizes and confidence intervals. Standard errors can be calculated using the equation β + (1.96 x SE)*

*Page 12, lines 262-263*

*Regression diagnostics were assessed for all models and found to be satisfactory.*

Model 1: Associations between number of purchases (exposure) vs diet quality (outcome)- unadjusted

**Model 1: Residual vs fitted plot**

- Residuals roughly centred around horizontal line-
- No strong suggestion of non-linearity

**Model 1: Normal Q-Q plot**

- Points fall along the diagonal line suggesting residuals are normally distributed

**Model 1: Leverage vs Residual Squared**

- Some outliers for leverage and residual are observed but no extreme outliners of concern

Model 2: Associations between number of purchases (exposure) vs diet quality (outcome)- Adjusted for age, gender, ethnicity and household SES

**Model 2: Residual vs fitted plot**

- Residuals roughly centred around horizontal line-
- No strong suggestion of non-linearity

**Model 2: Normal Q-Q plot**

- Points fall along the diagonal line suggesting residuals are normally distributed

**Model 2: Leverage vs Residual Squared**

- Some outliers for leverage and residuals are observed but no extreme outliners of concern

Model 3: Associations between weekly Purchasing Healthfulness Score (exposure) vs diet quality (outcome)- unadjusted

**Model 3: Residual vs fitted plot**

- No strong suggestion of non-linearity

**Model 3: Normal Q-Q plot**

- Points fall along the diagonal line suggesting residuals are normally distributed

**Model 3: Leverage vs Residual Squared**

- No extreme outliners of concern

Model 4: Associations between weekly Purchasing Healthfulness Score (exposure) vs diet quality (outcome)- Adjusted for age, gender, ethnicity and household SES

**Model 4: Residual vs fitted plot**

- No strong suggestion of non-linearity

**Model 4: Normal Q-Q plot**

- Points fall along the diagonal line suggesting residuals are normally distributed

**Model 4: Leverage vs Residual Squared**

- No extreme outliers of concern

Model 5: Interaction model- Associations between weekly purchasing healthfulness scores (exposure) vs diet quality scores (outcome) by household socioeconomic status (SES

**Model 5: Residual vs fitted plot**

- No strong suggestion of non-linearity

**Model 5: Normal Q-Q plot**

- Points fall along the diagonal line suggesting residuals are normally distributed

**Model 5: Leverage vs Residual Squared**

- No extreme outliners of concern

**Reviewers' comments:**

**Reviewer #1: This study reports the results of study using ecological momentary assessment to measure independent food purchases among adolescents and assesses the association with dietary quality via FFQ. A strength is the use of EMA methods, however the convenience sample is very small and I have doubts about the suitability of the sample for many of the analyses presented and some of the analytic approach.**

**1. The introduction and discussion are mostly well written and well referenced. Perhaps the discussion is a little on the long side and could be trimmed down using more concise language.**

In response to the reviewer’s comments, we have shorted the discussion section and used more concise language where possible.

The original Table 1 has also been moved to Supporting Material to shorten the main manuscript.

**Methods -
2. Line 48 - how were ppts recruited from these locations? Posters, email lists, announcements etc?**

Information has now been added to the manuscript to describe the recruitment process in greater detail.

Page 6, lines 114- 127

*Details about the study, including participant information sheets, were circulated to potential participants by announcements and student emails at the participating organisations. A researcher followed up in-person, when possible, by attending the school, college or youth group to provide further details and answer questions from the young people. Information (both written and verbal) was provided about the purpose and methods of the study. Written informed consent was obtained for all participants using an electronic consent form.* *Participants over the age of 16 years were able to provide consent to participate. Those aged under 16 years provided assent and parental consent was also sought for their participation.*

**3. Line 64 "where possible, member of research team..." may be useful to report as a %of total sample how many times research team were present**

Details describing the presence of the research team during data collection have been added to the manuscript. Please see excerpt with changes below.

*Page 7, lines 134 - 140*

*A member of the research team aimed to be present when the participants were completing the questionnaire and downloading the study app. On some occasions, restrictions on visitors entering schools and colleges during the COVID-19 pandemic prevented this happening. In these instances, participants were able to contact the research team via email if they experienced any difficulties. A researcher was present for 59% of app downloads.*

**4. Photographs were submitted for 23% of food entries, and photos / receipts were used to assess accuracy. What were the results of this checking process (ie how many of those photographs matched the ppts description of the food item?). Is there any other evidence to support that the participants were able to accurately and reliably categorise food items purchased?**

Thank you for this query. There were no additional methods available to ensure that participants were able to accurately and reliably categorise the food items they purchased. We recognise that this point is a limitation of the methods of this study and have now added this additional limitation to the strengths and limitations section in the discussion.

*Page 27, lines 512-519*

*While the purchasing data provided a more detailed description of purchases made by adolescents than many published studies, data about food brands, portion sizes and nutritional information were not collected. It is therefore possible that purchases could have been miscategorised in terms of healthfulness. Where photos were available (23% of purchases), the research team used these to cross-check that participants had accurately categorised their food purchases. This cross-check was not possible when photos were not provided*.

**5. Given the feasibility / acceptability results have not been published elsewhere, could the authors briefly summarise here? (Even if there are plans to publish this in future, a very high level summary would be useful)**

We thank the reviewer for this helpful comment and have now added a brief overview of the findings relating to feasibility/acceptability to the manuscript. Please see excerpt with the changes below.

*Page 8, lines 159-166*

*The app data collection methods were refined through feasibility testing (n=14) and a pre-pilot study (n=12) with young people to ensure the number of prompts as well as the wording and quantity of questions were acceptable (Shaw, 2023). Following the pre-pilot study, 83% of participants responded neutral, agree, or strongly agree to the statement, “I liked using the app.” However, 42% reported that there were too many notifications, leading to a reduction in the number of prompts to three per day in the full study.*

**6. The healthiness score tallied scores of -1 and 1 for non-adhering and adhering foods respectively. And then foods that could not be categorised were coded as 0 and included in this overall score. This approach essentially treats items of unknown "healthiness" as being halfway between the known values of -1 and 1. However, the more accurate treatment of these foods would be to code them as missing and not include them in the overall score. For example, a combination food "sandwich" might in reality fall in the "adhering" category. But defaulting to categorise it as "0" will pull the overall score downwards artificially. The opposite for a sandwich that would be accurately coded as "not adhering". I suggest the authors conduct the analyses coding these items as "missing" and present this as a sensitivity analysis, at the very least, otherwise replace analyses with the score recalculated treating uncategorised food as missing**.

A sensitivity analysis has been conducted as suggested by the reviewer. No major differences were observed with this approach. We have retained the original results in the main text, but details of this sensitivity analysis have been added to supporting material.

*Page 12, lines 247-252*

*In recognition that scoring food purchases categorised as ‘combination’ as ‘0’ may not be capturing the missing information associated with these foods, a sensitivity analysis was conducted with these purchases treated as missing. This sensitivity analyses showed no impact on the overall study findings. Full details of the sensitivity analysis can be found in the supporting materials.*

**Table 4 (Original Table 5)**

| **Exposure variable** | **Unadjusted β  (95% CI)** | **SE** | **p-value** | **Adjusted β  (95% CI) ^*^** | **SE** | **p-value** |
| --- | --- | --- | --- | --- | --- | --- |
| Number of weekly purchases | 0.00  (-0.04, 0.04) | 0.02 | 0.98 | 0.01  (-0.04, 0.04) | 0.02 | 0.78 |
| Weekly Purchasing Healthfulness Score (healthfulness units) | 0.48  (0.01, 0.96) | 0.24 | 0.05 | 0.36  (-0.15, 0.87) | 0.25 | 0.16 |
| ^*^Models adjusted for age, gender, ethnicity, and household SES.  Purchasing Healthfulness Scores closer to 1 indicate healthier purchasing | | | | | | |

**Table 4 When purchases coded as ‘0’ are treated as missing**

| **Exposure variable** | **Unadjusted β  (95% CI)** | **SE** | **p-value** | **Adjusted β  (95% CI) ^*^** | **SE** | **p-value** |
| --- | --- | --- | --- | --- | --- | --- |
| Number of weekly purchases | -0.00  (-0.04, 0.04) | 0.02 | 0.94 | 0.00  (-0.04, 0.05) | 0.02 | 0.85 |
| Weekly Purchasing Healthfulness Score (healthfulness units) | 0.43  (-0.01, 0.87) | 0.22 | 0.06 | 0.34  (-0.13, 0.81) | 0.24 | 0.16 |
| ^*^Models adjusted for age, gender, ethnicity, and household SES.  Purchasing Healthfulness Scores closer to 1 indicate healthier purchasing | | | | | | |

**7. Line 151 - I'm not familiar with the term "test for trend" or how it retains statistical power (which test is this one replacing due to small numbers?)**

We thank the reviewer for the ability to detail the meaning behind this term. In order to preserve statistical power, these relationships were tested using Pearson’s correlation with the underlying continuous variable rather than the categorical variable presented. The text has now been amended to provide clarity.

*Page 11, lines 236-240*

*Differences in diet quality scores and purchasing outcomes according to demographic characteristics were assessed using unpaired t-tests and Mann-Whitney rank-sum tests. In order to retain statistical power, a Spearman’s correlation test was conducted for characteristics measured using an underlying continuous variable (age, IMD, and household SES).*

**Results-
8. What was the justification for the sample size recruited? Was an a priori power calculation conducted? Rationale is needed. In particular, I'm doubtful that the N provides sufficient power for the interaction test (maybe on the low side to address aim 1, even). Further, statistical testing of many of the subgroup differences presented in table 4 is not appropriate given the very small sample size. Further, in text, these results are described and statements about purchasing being "higher" in one group compared to another are based on comparison of the raw numbers. Despite p values being reported (which I don't think is appropriate for many of the presented analyses), they don't appear to have been used as a criterion for determining where differences lie. I'd suggest being very clear in the text that these comparisons are not supported by statistical tests due to small numbers within subgroups.**

We agree with the reviewer that this study had a small sample and that interpretation of the results should therefore be cautious. As this was a pilot study, we did not conduct an apriori sample size calculation. We are aware that it is unhelpful to describe results as ‘statistically significant’ or ‘statistically non-significant’ depending on which side of an arbitrary cut-off of 0.05 the P-value falls ([https://www.nature.com/articles/d41586-019-00857-9](https://eur03.safelinks.protection.outlook.com/?url=https%3A%2F%2Fwww.nature.com%2Farticles%2Fd41586-019-00857-9&data=05%7C02%7CSarah.shaw%40mrc-epid.cam.ac.uk%7C427e8c0f03f9448984b308dd8d560c72%7C49a50445bdfa4b79ade3547b4f3986e9%7C1%7C0%7C638822122921298184%7CUnknown%7CTWFpbGZsb3d8eyJFbXB0eU1hcGkiOnRydWUsIlYiOiIwLjAuMDAwMCIsIlAiOiJXaW4zMiIsIkFOIjoiTWFpbCIsIldUIjoyfQ%3D%3D%7C0%7C%7C%7C&sdata=YRotUlA2ipYfBGbm%2BjEwnM7DDL8rl2hU%2FQBc281hPbE%3D&reserved=0)). We have conducted statistical tests for clarity but base our interpretation on effect sizes rather than emphasising P-values. We have added a sentence to the methods section of the manuscript to ensure this approach is clearly described. Please see the excerpt below.

Page 12, lines 258-262

*Due to the pilot nature of the study, the results were interpreted based on effect sizes rather than emphasising statistical significance; this stance is in line with current statistical thinking on reporting findings from quantitative studies in medicine (Amrhein et al. 2019).*

**9. Given the study used a convenience sample, what was the rationale behind presenting adjusted analysis (including demographics that were unequally distributed across levels of the key outcome and exposure variables) as sensitivity analyses? It seems that failing to control for these variables is not optimal treatment of the data. I would encourage authors to present adjusted analyses as the primary analyses, and then report the unadjusted analyses as sensitivity analyses.**

We agree with the reviewer that the most important analyses are those adjusting for confounders. However, in line with convention we present unadjusted analyses followed by adjusted analyses. We do not describe either of these as ‘sensitivity analyses’ in the text but rather present the unadjusted and adjusted analyses in an order that will aid readers’ understanding.

To ensure the emphasis is placed on the adjusted findings, we have removed the unadjusted results from the abstract.

Page 2, abstract lines 38-46

*Over seven days, 552 food/drink items were purchased on 253 food purchasing occasions by 80 participants. The majority of purchases (n=329, 60%) were coded as ‘not adhering’ to the UK Eatwell Guide, 32% were coded as ‘adhering’ and 9% fell between these categories and were coded as ‘combination’’. In fully adjusted models, adolescents who made healthier food purchases tended to have better quality diets (β 0.36, (95%CI -0.15, 0.87) p=0.16). Interaction models showed purchasing healthfulness was more strongly associated with diet quality among young people of lower SES (p=0.01).*

**10. In tables and figures presenting healthfulness scores, it would be useful to specify as a reminder that scores closer to 1 indicate a healthier score, and closer to -1 indicate less healthy. This will assist readers viewing these elements out of context.**

We have added the following sentence to each table presenting the healthfulness score (Tables 3, 4, and 5)

*Purchasing Healthfulness Scores closer to 1 indicate healthier purchasing*

**11. Line 228 "this relationship remained when.." this is a bit confusing given there was no significant relationship in the first place, suggest amending text to "there was also no significant relationship between x and y when..."**

In line with point 8 above, we have reduced the focus on p values throughout the manuscript and the text has been amendment to reflect effect size throughout. P-values are presented for clarity.

Page 19, lines 337-341

*Table 4 presents the results of linear regression analyses, testing the association between food purchasing and diet quality. Unadjusted models showed no notable relationship between number of weekly food purchases and diet quality. There was also no notable relationship when models were adjusted for age, gender, ethnicity, and household SES.*

**12. The conventional criteria for statistical significance is p less than .05, not equal to .05. Please amend the text in the results at line 232, Abstract, and Discussion accordingly.**

In line with point 8 above, we have reduced the focus on p values throughout the manuscript and the text has been amendment to reflect effect size throughout. P-values are presented for clarity.

**13. The information presented in Figure 2 would be easily conveyed in text, so I would suggest deleting this figure for brevity.**

We thank the reviewer for this suggestion. Figure 2 has now been removed from the manuscript because it did not add meaningfully to the paper. A text description has been added to the main text. Please see excerpt below.

Page 15, lines 290-295

*Similar purchasing patterns were shown between adolescents from high and low SES households. For adolescents from high SES households, 214 food items (60%) were coded as ‘not adhering’, 112 (31%) were coded as ‘adhering’ and 32 (9%) were coded as combination foods. For adolescents from low SES households, 115 food items (59%) were coded as ‘not adhering’, 63 (32%) were coded as ‘adhering’ and 16 (8%) were coded as combination foods.*

**14. I wasn't sure whether the results reported in text and in figure 3 were based on regression analyses adjusting for all other covariates or not. Please ensure this is clear- either way, from the methods it sounds as though both adjusted and unadjusted were run, but at present only one set of analyses are reported in the results.**

We have added further detail to the text and the Figure 2 (previously Figure 3) caption to make it clear that these analyses are adjusted for age, gender, ethnicity and household SES.

*Page 21, Figure 2 caption*

*Figure 2 Associations between diet quality scores and weekly purchasing healthfulness scores by household socioeconomic status (SES), based on linear regression analysis adjusted for age, gender, ethnicity, and household SES*

Page 20, line 353-360

*Adding the interaction term to the regression models showed that the association between Weekly Purchasing Healthfulness Score and diet quality was stronger among participants with low household SES compared to those with high household SES (p for interaction=0.01) (Fig 3).Stratified analyses, adjusted for age, gender, ethnicity and household SES, showed a positive relationship between the healthfulness of weekly purchases and diet quality for adolescents from households of lower SES (β 1.04 SDs/healthfulness unit, (95%CI 0.26, 1.81) p=0.01).*

**Discussion
15. Line 372 "because of uncertainty regarding who made no recordings" - I'm struggling to grasp what this sentence means - I note that the following sentence provides further detail, but I'd suggest replacing them both with the following wording or similar: "...because it could not be determined whether participants who did not record any purchases did so because they did not make any independent food purchases or because they did not adhere to the study protocol"?**

We thank the reviewer for this suggestion and have made these changes to improve readability.

*Page 27, lines 507-510*

*In this study, it was not possible to determine the completeness of the purchasing data. Participants who did not record any purchases may have not made any independent food purchases. Alternatively, they may not have adhered to the study protocol.*
