# Supplementary material for: The association between adolescents’ independent food purchasing and dietary quality differs by socioeconomic status: Findings from a pilot study
Source: PLoS One. 2025 Sep 4;20(9):e0312903. doi: 10.1371/journal.pone.0312903 (PMC12410734; doi:10.1371/journal.pone.0312903)
Supplement: Attachment — Submitted filename: Response to review_PLOS_17July25_F.docx [file pone.0312903.s005.docx]

Editor-in-chief

PLOS One

17^th^ July 2025

Dear Editor,

**RE: PONE-D-24-45297
What role do adolescents’ independent food purchasing choices play in their dietary quality?**

We thank the editorial team and reviewer for their helpful review of our manuscript and for providing us with valuable feedback. Below, we have reproduced the reviewer’s comments in **bold** and added our responses in regular font indicating how we have altered the paper to address the comments. Excerpts from the manuscript are also provided in *italicised* text with corresponding page and line numbers for the tracked manuscript, and text changes highlighted in red font.

For clarity, we have also directly responded to the reviewer’s comments on the word document with tracked changes. As requested, a clean version with the amendments has also been attached.

We look forward to hearing the outcome of the peer review process.

Yours sincerely,

The authors

Journal requirements

1. **Please review your reference list to ensure that it is complete and correct. If you have cited papers that have been retracted, please include the rationale for doing so in the manuscript text, or remove these references and replace them with relevant current references. Any changes to the reference list should be mentioned in the rebuttal letter that accompanies your revised manuscript. If you need to cite a retracted article, indicate the article’s retracted status in the References list and also include a citation and full reference for the retraction notice.**

We have reviewed the reference list to ensure it is complete and correct. We have also changed the referencing style to Vancouver, in line the with journal requirements. Changes have been made to references 14 and 15 to ensure they are correctly formatted.

References

*14.	Locatienet. Locatienet. 2023. [Available from:* [*https://www.locatienet.com*](https://www.locatienet.com)*.]*

*15.	Tripzoom. Tripzoom. 2023. [Available from:* [*https://en.tripzoom.nl/*](https://en.tripzoom.nl/)*.]*

2. **Thank you for stating the following in the Competing Interests section:**

**[SS, SC, CC, DS, MB have no conflicts of interests to declare. CV has a non-financial research collaboration with a UK supermarket chain. The study described in this manuscript is not related to this relationship.].**

**Please confirm that this does not alter your adherence to all PLOS ONE policies on sharing data and materials, by including the following statement: "This does not alter our adherence to PLOS ONE policies on sharing data and materials.” Please include your updated Competing Interests statement in your cover letter; we will change the online submission form on your behalf.**

As requested, we have made the edits to our Competing Interests statement to confirm it does not alter our ability to adhere with PLOS ONE policies on sharing data. We have included the edited statement below, we would be grateful if this could be updated in the online submission system.

Edited Competing interests statement

*SS, SC, CC, DS, MB have no conflicts of interests to declare. CV has a non-financial research collaboration with a UK supermarket chain. The study described in this manuscript is not related to this relationship. This does not alter our adherence to PLOS ONE policies on sharing data and materials.*

Reviewer comments

1. **Title: Kindly turn this into a statement**

We thank the reviewer for this suggestion. We have rephrased the title into a statement. The title is now:

Title

*The association between adolescents’ independent food purchasing and dietary quality differs by socioeconomic status: Findings from a pilot study*

2. **Abstract line 50-53: The statements contradicts each other, please verify**

We thank the reviewer for pointing out that our description of the findings lacked clarity. We acknowledge that presenting the findings using different directional terms may have made interpretation difficult. We have revised the abstract to improve clarity and ensure they are more easily understood by the reader.

Abstract lines 41-53

*Over seven days, 552 food/drink items were purchased on 253 food purchasing occasions by 80 participants. The majority of purchases (n=329, 59%) were coded as ‘not adhering’ to the UK Eatwell Guide, 32% were coded as ‘adhering’ and 9% fell between these categories, being coded as ‘combination’.* *The healthfulness of food purchases did not differ between adolescents from low- and high-SES households. Across all adolescents, 39% reported that their food and drink purchases were snacks and these were less healthful than purchases made for main meals. Fully adjusted regression models showed that adolescents who made less healthy food purchases tended to have poorer quality diets (β 0.36, (95%CI -0.15, 0.87) p=0.16). Interaction models showed that less healthy purchasing was more strongly associated with poorer diet quality among young people of lower SES than those of higher SES (p=0.01).*

3. **Keywords: Kindly add at least two extra keywords**

As suggested by the reviewer, we have added two additional keywords to describe this study. The new keywords are listed below.

Keywords:

*Adolescents; dietary behaviours; food purchasing; diet quality; socio-economic status; dietary inequalities*

4. **Introduction Lines 84-88: Do you have a recent study of adolescents food intake?**

Thank you for this helpful comment. Few studies have provided a detailed assessment of adolescent food purchasing behaviours. The 2015 report we reference offers detailed insights into the types of foods adolescents purchase for themselves at school lunchtimes. We have added a sentence to the introduction to better emphasise the lack of published evidence describing adolescents’ independent food purchases. In the discussion, we also highlight how our study addresses an important gap in the literature.

Introduction lines 81-87

*Few studies have provided detailed information about the types of foods adolescents purchasing for themselves. A 2015 report from Food Standards Scotland showed that the majority of independent food choices made by adolescents outside of the home and school at lunchtimes are high in fat, sugar and salt; chips (fries), sandwiches, sugary soft drinks and energy drinks were some of the most frequently purchased items (9).*

Discussion lines 380-384

*This study also provides insights into the types of foods adolescents purchase for themselves and offers a more nuanced understanding than previous studies which have reported adolescent purchasing of only specific food items (e.g. sugar-sweetened beverages) or unspecific categories such as junk food or snack food (30, 31).*

5. **Methods line 176: Kindly check if this correct**

We have checked to ensure the name of the survey is correct. We have added a reference for the survey.

Methods line 175-176

*These questions have been used in the UK-based ‘What about YOUth?’ Survey (22)*

6. **Methods line 198-205: It is good that you have the supplementary File. Kindly add just an example of the food items classified as adhering and not adhering in the main file**

Thank you for highlighting this omission. We have now provided examples in the text of each categorisation.

Methods 198-205

*Food and beverage items which are included as part of the main food groups in the Eatwell Guide and those that individuals are encouraged to eat more were categorised as ‘adhering’ (e.g. fruit, non-sugar sweetened drinks, bread). Foods high in fat, sugar and salt that fall outside the main food groups in the Eatwell guide and which individuals are encouraged to limit were categorised as ‘not-adhering’ (e.g. crisps, sugar-sweetened beverages, confectionary). Foods that could not be classified within either of these groups were classified as ‘combination’ foods (e.g. sandwich/wraps, hot drinks (not tea or coffee)). Supporting Materials Table 1 shows the food purchases recorded by participants and how they were classified in terms of healthfulness*

7. **Methods 223: Indicate the name of the FFQ**

A specific name is not available for the FFQ but we have provided more detail about the tool and added the reference where the FFQ is first described in the manuscript.

Methods 223-224

*Using a 20-item FFQ that was designed to assess diet quality among UK adolescents in population level studies (11), participants recorded how frequently they consumed each food group over the previous month.*

8. **Table 1 : Kindly indicate the meaning of ***

Thank you for highlighting this omission. We have added the following text to the footnote of Table 1.

Footnote of Table 1

** Other ethnicities: Black African, Bangladeshi, Pakistani, Chinese, Afghan, British Indian, British Punjabi, Arabic, British Asian, Turkish, Mixed*

9. **Table 1: Were the 28 with incomplete data significantly different from the 80?**

We have tested for differences between those with and without purchasing data. We have added these findings to Table 1. We also added a description of these findings to the participant characteristics paragraph in the results section. We have also added a comment to the strengths and limitations sub-section of the discussion section which describes the implications of these differences.

Table 1 Characteristics of study participants

| **Characteristics** | **Participants with purchasing and diet data (n=80)** | **Participants without purchasing data (n=28)** | **p-value** |
| --- | --- | --- | --- |
| **Gender, n (%)** Boy Girl Not provided | 19 (24) 55 (69) 6 (7) | 14 (50) 13 (46) 1 (3) | 0.03 |
| **Age, median (IQR) (years)** | 17 (15,17) | 14 (13,17) | <0.01 |
| **Age, n (%) (years)** 11-13 years 14-16 years 17-18 years | 8 (10) 25 (31) 47 (59) | 9 (32) 10 (36) 9 (32) | <0.01 |
| **Ethnicity, n (%)** White  Other ethnicities* | 63 (79) 17 (21) | 25 (89) 3 (11) | 0.22 |
| **Area-level SES (IMD),  n (%)** 1-5 (most deprived) 6-10 (least deprived) Missing | 30 (37) 34 (43) 16 (20) | 12 (43) 13 (46) 3 (11) | 0.92 |
| **Household-level SES (FAS), n (%)** Low (0-7) High (8-14) Missing | 24 (30) 54 (68) 2 (2) | 5 (18) 23 (82) 0 (0) | 0.21 |
| ^* Other ethnicities: Black African, Bangladeshi, Pakistani, Chinese, Afghan, British Indian, British Punjabi, Arabic, British Asian, Turkish, Mixed^ | | |  |

Results lines 277-278

*Boys (p=0.03) and younger adolescents (p<0.01) were more likely to have no purchasing data.*

Discussion lines 476-482

*Boys and younger adolescents were more likely to report no food purchases. Of those who did record purchases, girls and older adolescents tended to record higher quantities of food purchases during the study period. These patterns suggest that the absence of recorded purchases may reflect genuine lack of independent food purchasing rather than missing data. However, it is also possible that some participants who reported no purchases did not fully adhere to the study protocol.*

10. **Results Lines 290-292: These are above 100% please check**

Thank you for highlighting this error which occurred due to rounding. We have now checked the percentages to ensure they equal 100%. We have changed these findings in the main text and in the abstract.

Results Lines 290-292

*The majority of purchases (n= 329, 59%) were coded as ‘not adhering’ to the UK Eatwell Guide, 175 food items (32%) were coded as ‘adhering’ and 48 (9%) were coded as combination foods.*

11. **Results line 294-298: This is very important but contradicts what is presented in the abstract. Between 60% and 59% is only 1% difference ie all adolescents are not eating well. This has implications that needs to be reflected on.**

Thank you for pointing out the lack of clarity in our abstract. We did not intend to imply that our findings demonstrated differences in the healthfulness of purchasing between young people from different SES backgrounds. To more accurately reflect the study results, we have revised the abstract and added a clarifying sentence.

Abstract Lines 41-50

*The majority of purchases (n=329, 59%) were coded as ‘not adhering’ to the UK Eatwell Guide, 32% were coded as ‘adhering’ and 9% fell between these categories were coded as ‘combination’.* *The healthfulness of food purchases did not differ between adolescents from low- and high-SES households. Across all adolescents, 39% reported that their food and drink purchases were snacks and these were less healthful than purchases made for main meals. Fully adjusted regression models showed that adolescents who made less healthy food purchases tended to have poorer quality diets (β 0.36, (95%CI -0.15, 0.87) p=0.16).*

12. **Results line 309: This is also vital; because it indicates that they are having their main meals at home and are similar to adults as their snacks are also unhealthy**

Thank you for highlighting the importance of this finding. To emphasise the importance of this finding, we have added an additional sentence to the abstract. The implications of this findings are highlighted in the discussion.

Abstract Lines 41-50

*The majority of purchases (n=329, 59%) were coded as ‘not adhering’ to the UK Eatwell Guide, 32% were coded as ‘adhering’ and 9% fell between these categories were coded as ‘combination’.* *The healthfulness of food purchases did not differ between adolescents from low- and high-SES households. Across all adolescents, 39% reported that their food and drink purchases were snacks and these were less healthful than purchases made for main meals. Fully adjusted regression models showed that adolescents who made less healthy food purchases tended to have poorer quality diets (β 0.36, (95%CI -0.15, 0.87) p=0.16).*

Lines 461-470

Snack purchases made up the largest proportion of purchases by adolescents in the current study and tended to be less healthful. This finding is consistent with research from the Netherlands which showed adolescents mainly purchased snack food items from supermarkets close to school and spent, on average €2.30 (~USD 2.45) (45). Qualitative research shows that adolescents favour quick, single-serve, low-cost snack options (46). Future research and food policy development would benefit from targeting the availability of healthier snack options that are affordable and appealing to adolescents. These healthier snack options should be available in the food outlets adolescents regularly visit and offer a possibility for new product development.

13. **Results lines 361-364: Please be careful how you interpret this. It could just mean that they are purchasing less than those from high SES HHs. You reported this in the food purchasing section**

Thank you for this comment. Throughout the manuscript we have rephrased our findings to ensure the results are consistently described.

Results Lines 354-358

*Stratified analyses, adjusted for age, gender and household SES, showed that less healthy weekly purchasing was associated with poorer diet quality among adolescents from households of lower SES (β 1.04 SDs/healthfulness unit, (95%CI 0.26, 1.81) p=0.01).*

As highlighted in the methods sections (Lines 210-218) the purchasing scores take into consideration the number of purchases made by each adolescents. In this stratified analysis, we are therefore confident that the results are not a reflection of a difference in the number of purchases being made.

Methods Lines 210-218

*To create Purchasing Healthfulness Scores, foods classified as ‘adhering’ were allocated the score of ‘+1’, the ‘combination’ classification was allocated the rating of ‘0’ and the ‘non-adhering’ foods were allocated the rating of ‘-1’. Food scores were subsequently tallied for each food purchasing occasion and then divided by the total number of food/drink items purchased as part of that food purchasing occasion. The same process was used to calculate a Weekly Purchasing Healthfulness Score for each participant; food scores were tallied for the entire week and then divided by the total number of food/drink items purchased over the week.*

14. **Discussion Line 391: Your results did not support this.**

This sentence describes findings from previous research not our own research. To improve clarity in the manuscript, we have added further text to ensure it is clear to readers that this description relates to prior research.

Discussion Line 391-394

*Disparities in dietary behaviours between socioeconomic groups are well documented in the literature. Previous research has shown that adolescents from disadvantaged backgrounds tend to have poorer quality diets, with higher intakes of energy-dense, nutrient-poor foods and lower intakes of fruits and vegetables (32-34).*

15. **Discussion line 404: Kindly focus on adolescents regardless of their SES are eating unhealthy snacks and their implications for their future dietary patterns and health**

Thank you for this suggestion. We have rephrased the start of this paragraph to reflect the food purchasing behaviours of all adolescents.

A separate discussion point, relating to snack purchases, can be found later in the discussion section.

Discussion lines 404 - 423

*Despite this research showing that on average all adolescents make more less healthy than healthy food purchases, this practice was shown to be most strongly associated with the dietary quality of adolescents with lower SES. One potential explanation for this finding is that independent food purchases make up a larger proportion of the overall diets of adolescents from more deprived backgrounds. Since these purchases tend not to conform to healthy eating guidelines, they are likely to reduce the overall quality of these young people’s diets. Another driver of this difference may be that adolescents from less affluent backgrounds are not exposed to the same health-promoting foods at home as those living in more affluent households. Previous research conducted among Scottish families with teenagers found that even though health is valued when making food choices, families from lower SES were driven to make choices that ensured all family members were fed in a quick and acceptable way (Wills et al. 2011). Backett-Milburn et al (2006) described how competing priorities within families with lower-incomes means nutrition and health fall below other factors such as affording everyday essentials, safety and worries about children engaging in other risky behaviours such as drugs, alcohol and smoking (Backett-Milburn et al. 2006).*

Discussion lines 461-470

*Snack purchases made up the largest proportion of purchases by adolescents in the current study and tended to be less healthful. This finding is consistent with research from the Netherlands which showed adolescents mainly purchased snack food items from supermarkets close to school and spent, on average €2.30 (~USD 2.45) (45). Qualitative research shows that adolescents favour quick, single-serve, low-cost snack options (46). Future research and food policy development would benefit from targeting the availability of healthier snack options that are affordable and appealing to adolescents. These healthier snack options should be available in the food outlets adolescents regularly visit and offer a possibility for new product development.*
